# Overview of Recent Advances in Rare-Earth High-Entropy Oxides as Multifunctional Materials for Next-Gen Technology Applications

**DOI:** 10.3390/molecules30051082

**Published:** 2025-02-27

**Authors:** Stjepan Šarić, Jelena Kojčinović, Dalibor Tatar, Igor Djerdj

**Affiliations:** Department of Chemistry, Josip Juraj Strossmayer University of Osijek, Cara Hadrijana 8/A, HR-31000 Osijek, Croatia; stjepan.saric@kemija.unios.hr (S.Š.); jbijelic@kemija.unios.hr (J.K.); dtatar@kemija.unios.hr (D.T.)

**Keywords:** high-entropy oxides, rare-earth elements, configuration entropy, catalysis, sustainability, CO oxidation, CO_2_ hydrogenation, hydrogen production, optoelectronics

## Abstract

Rare-earth high-entropy oxides are a new promising class of multifunctional materials characterized by their ability to stabilize complex, multi-cationic compositions into single-phase structures through configurational entropy. This feature enables fine-tuning structural properties such as oxygen vacancies, lattice distortions, and defect chemistry, making them promising for advanced technological applications. While initial research primarily focused on their catalytic performance in energy and environmental applications, recent research demonstrated their potential in optoelectronics, photoluminescent materials, and aerospace technologies. Progress in synthesis techniques has provided control over particle morphology, composition, and defect engineering, enhancing their electronic, thermal, and mechanical properties. Rare-earth high-entropy oxides exhibit tunable bandgaps, exceptional thermal stability, and superior resistance to phase degradation, which positions them as next-generation materials. Despite these advances, challenges remain in scaling up production, optimizing compositions for specific applications, and understanding the fundamental mechanisms governing their multifunctionality. This review provides a comprehensive analysis of the recent developments in rare-earth high-entropy oxides as relatively new and still underrated material of the future.

## 1. Introduction

The development of advanced materials, as one of the most important focuses in scientific research, roughly means the discovery of substances with adaptable and enhanced physico-chemical properties. “Transformation” in material science was introduced by the discovery of high-entropy alloys, which have offered opportunities to manipulate the phase stability of solid solutions through configurational entropy [1]. This approach represents an advancement in the material design and understanding of complex material systems. High-entropy materials, including non-metallic compounds such as oxides [2], fluorides [3], sulfides [4], nitrides [5], etc., are distinguished by their ability to form single-phase multielement structures. This material category exhibits higher levels of mixing entropy (*S*_mix_) or configurational entropy (*S*_config_) than their constituent elements, resulting in crystal structures distinct from those of the latter. The thermodynamic stability of these materials is governed by the Gibbs energy of mixing at high temperatures, which enables the formation of single-phase solid solutions when the entropy contribution to the crystal system outweighs the enthalpy of mixing. The main criteria identifying high-entropy materials have been expanded to include systems containing five or more elements in concentrations ranging from 5 to 35% [6], as well as those with configuration entropy values exceeding by 1.5 times the universal gas constant from the Boltzmann entropy equation [7]. These definitions allow for the inclusion of a wide range of materials with unique properties and potential applications.

In the field of high-entropy oxides (HEOs), a breakthrough was first achieved by Rost et al. [2], who demonstrated the successful incorporation of five distinct cations into a single-phase oxide system. This achievement introduced the concept of entropy-stabilized oxides, which marked a fundamental shift in the understanding of phase stabilization in solid solutions. Their work demonstrated that by increasing the number of metal cations in the cationic sublattice, the system’s configurational disorder is significantly enhanced, resulting in stabilization effects similar to those observed in multi-element alloys. From a thermodynamic perspective, the configurational entropy of multi-element oxides can be derived using the Boltzmann entropy equation, where the number and relative proportions of distinct cations and anions contribute to the overall entropy. The parameter *S*_config_ provides a quantitative measure of the material’s complexity, with the entropy value increasing as a function of the number of cations in the system. In typical HEOs, the assumption is that the oxide ion serves as the sole anionic species within the lattice, simplifying the structural analysis.

The selection of suitable components for the formation of single-phase HEOs depends on several factors, including mixing enthalpy, ionic radius, oxidation state, and coordination number of the cations. The main idea is to achieve the so-called “cocktail effect”, which indicates that pristine metal oxides have different properties compared with the ones they acquire while in high-entropy form. The mixing enthalpy exerts an influence only under specific conditions, such as in solid-state or mechanochemical synthesis, where appropriate metal oxides serve as precursors. However, it is important to note that the mixing enthalpy is less applicable in solution-based syntheses using non-oxide precursors [8], such as alkoxides or nitrates. For optimal HEO formation, the mixing enthalpy value should ideally be close to zero, which is particularly relevant in solution-based methods. Extremely positive or negative mixing enthalpy values, typical for certain metal oxides, can pose challenges to achieving single-phase high-entropy compounds. These cases may require higher temperatures to facilitate phase transitions and mixing. Another parameter is the ionic radius of the metal cations, which significantly influences the formation of single-phase structures. Selecting cations with similar ionic radii is crucial for obtaining a single-phase structure, particularly in sodium chloride or fluorite crystal configurations [9]. In such systems, isovalent metal cations occupy equivalent positions, while anions maintain the lattice’s overall electrical neutrality [10]. The oxidation state of the metal cations is equally critical for determining the type of crystal structure. For instance, in cubic oxides with a NaCl structure, metal cations typically exhibit a +II oxidation state, while in fluorite structures, cations often have a +IV oxidation state. This flexibility in the oxidation state allows for the formation of single-phase HEOs with varying crystal structures, providing a versatile platform for designing materials with tailored properties [11]. High-entropy materials such as high-entropy alloys, metal (oxy)hydroxides, oxides, fluorides, etc., are widely used materials for water electrolysis. Their structural stability, compositional flexibility, and synergy between elemental components has given a new insight into green hydrogen production by water electrolysis, reported by Xu et al. [12]. The main goal is to reduce the carbon footprint using high-entropy materials as water-splitting catalysts to produce hydrogen as a cleaner alternative by water electrolysis [12]. A variety of physical and chemical approaches have been developed to address the thermodynamic and kinetic requirements for synthesizing HEOs. Achieving homogeneous single-phase solid solutions with uniform elemental distribution requires the precise control of synthesis methods and conditions to optimize material properties such as particle size, phase structure, and chemical composition. Traditional solid-state synthesis methods, which involve mixing metal salts or oxides, are commonly employed for producing HEO powders. However, these approaches typically require high temperatures [13], which can be a limitation. In contrast, low-energy solution-based methods are more interesting due to their ability to synthesize HEO nanoparticles with diverse compositions and crystal structures. These include co-precipitation [14], solvothermal [15] and hydrothermal synthesis [14], etc. Another way to synthesize HEO nanoparticles is mechanochemistry, specifically ball milling, which is achieved by applying external mechanical energy [10]. Such innovative methods offer enhanced control over key synthesis parameters, enable the development of materials with desirable properties, and minimize the high-temperature requirements associated with conventional approaches.

Rare-earth-based HEOs represent a rapidly emerging class of multifunctional materials with potential for next-generation technological applications. Even though research in this field remains in its early stages and the number of reported studies is still limited, preliminary findings suggest that their unique properties are more than promising, as these materials may offer transformative advantages over conventional oxides. Due to the limited number of studies in this field, this review main goal is to identify existing challenges, indicate the most important research gaps, and establish a framework for future investigations.

## 2. Rare-Earth Elements

### 2.1. Properties and Significance

Rare-earth elements are commonly classified into two categories: the lanthanide series and the elements scandium and yttrium. The lanthanides, comprising elements with atomic numbers ranging from 57 (lanthanum) to 71 (lutetium), represent a distinct group characterized by their unique atomic structure, which sets them apart from other elements, while they share some similarities with actinides. The lanthanides exhibit several consistent characteristics [16]. Physically, their properties remain uniform across the series. In crystalline compounds, they typically display a trivalent oxidation state, although divalent and tetravalent states are also observed in some cases. These compounds often possess coordination numbers greater than VI, with a general trend of decreasing coordination numbers across the series [17]. Furthermore, the lanthanides exhibit a strong affinity for highly electronegative elements, such as oxygen or fluorine [17]. The unique behavior of the lanthanides can largely be attributed to their electronic configurations. The neutral lanthanides generally exhibit an electronic configuration of 4*f*^n+1^, 5*s*^2^, 5*p*^6^, and 6*s*^2^, where the progressive filling of the 4*f* orbitals defines their chemical properties [17]. The formation of the characteristic trivalent cations occurs through the loss of one 4*f* electron and two 6*s*^2^ electrons. In certain instances, a small energy difference between the 4*f* and the 5*d* orbitals allows for electron transitions, leading to the formation of tetravalent ions, such as Ce^4+^, or divalent ions, such as Sm^2+^, Yb^2+^, and Eu^2+^. One notable phenomenon observed across the lanthanide series is “lanthanide contraction”, a gradual decrease in the ionic radius from La^3+^ (1.06 Å) to Lu^3+^ (0.85 Å) [18]. This contraction arises due to the incomplete shielding of the increasing nuclear charge by the 4*f* orbitals, which results in a stronger effective nuclear attraction. This contraction significantly influences the bonding behavior and coordination chemistry of these ions. Rare-earth elements play an indispensable role in modern technology due to their exceptional magnetic [19], phosphorescent [20], and catalytic properties [21].

### 2.2. Properties and Application of Ceria

Cerium dioxide, commonly known as ceria (CeO_2_), and its doped variants have been the focus of intensive research over the last several decades due to their remarkable properties, including structural stability, electronic and ionic conductivity, elastic behavior, and catalytic efficiency [22]. Ceria is mostly used as a base model for the synthesis of high-entropy rare-earth oxides, since the cerium atoms have a tendency to quickly and efficiently change their oxidation state from +III to +IV and vice versa. Guided by this statement, ceria-based high-entropy oxides hold a unique ability for catalytic reactions due to the high oxygen mobility and effortless formation of oxygen vacancies. Another huge advantage of cerium-based materials is that doping ceria with rare-earth elements or transition metals, enhances the physicochemical properties of such high-entropy materials. For example, by incorporating Zr into the structure of pure cerium dioxide, the oxygen storage/release capacity (OSC) of CeO_2_ is increased significantly [23]. These materials have proven highly suitable for a range of industrial applications, owing to their exceptional functional attributes and chemical inertness, which make them compatible with both inorganic and biological systems [24]. Typically, they adopt a fluorite-type crystal structure, characterized by cubic symmetry within the *Fm*-3*m* space group. In this configuration, the cerium atoms occupy the 4*a* lattice sites (0, 0, 0), while the oxygen atoms reside in the 8*c* positions (1/4, 1/4, 1/4) [25]. The fluorite structure demonstrates stability across broad temperature ranges and accommodates significant variations in oxygen stoichiometry, manifesting as oxygen vacancies. Upon reduction to compositions approximating CeO_1.7−1.8_, a disordered non-stoichiometric phase associated with the fluorite structure emerges [26]. Further increases in oxygen deficiency lead to the development of superstructures, arising from the ordered arrangement of vacancies within the lattice [27]. Although fluorite structures may appear simplistic, they exhibit a high degree of complexity due to their ability to incorporate substantial concentrations of lattice defects, particularly oxygen vacancies [28]. The structural stability of fluorites under varying temperature and stoichiometry conditions often obscures the underlying local atomic arrangements, which are critical to their behavior in complex materials with practical applications [29]. Extrinsic doping is a common strategy for modifying CeO_2_ to enhance its properties [24]. Trivalent dopants are often used as acceptors, creating oxygen vacancies in a 2:1 ratio to maintain charge neutrality. Dopants such as samarium [30] and gadolinium [31] are notable for their ability to achieve high ionic conductivity, which makes them ideal for such applications. In contrast, divalent dopants create a higher density of oxygen vacancies but generally exhibit lower ionic conductivity [32], which limits their practical use. Even without extrinsic doping, CeO_2_ can host intrinsic defects, particularly through the partial reduction of Ce^4+^ to Ce^3+^. This behavior leads to a combination of ionic and electronic conductivity [33], which becomes increasingly significant under high temperatures and low oxygen partial pressures. Non-stoichiometric CeO_2_ exhibits exceptional catalytic activity, particularly in automotive three-way catalysts, where a precise oxygen/fuel ratio control is critical [34]. Its ability to store and release oxygen efficiently, known as oxygen storage capacity (OSC), has also made it a key material in solar-driven thermochemical water-splitting technologies for clean energy production [35]. Furthermore, doping with tetravalent cations such as zirconium or hafnium enhances the reducibility of CeO_2_, improving its OSC and lowering the operational temperatures for applications like exhaust gas treatment and thermochemical processes [36].

#### Ceria Electronic Structure and Defect Chemistry

Ceria exhibits a complex electronic structure defined by two energy gaps: one between the 2*p* and 4*f* orbitals, and the other between the 2*p* and 5*d* orbitals. Oxygen vacancies emerge as fundamental intrinsic defects when oxygen atoms leave their lattice positions. These defects create a doubly charged vacancy, which induces a reduction in the oxidation state of the nearby cerium ions from +IV to +III [37]. The formation of such vacancies introduces significant local lattice distortions, where the neighboring oxygen atoms are pulled inward, and the cerium atoms shift outward, generating strain within the crystalline matrix [37]. The stoichiometric variation in ceria is governed by the temperature and oxygen partial pressure, with defect concentrations directly influencing the material’s properties. The presence of oxygen vacancies enhances ionic conductivity by facilitating the diffusion of oxygen ions through the lattice, a process critical for solid-state ionic devices. Moreover, these vacancies play a pivotal role in ceria’s catalytic activity, enabling the material to dynamically store and release oxygen. This oxygen storage capacity is central to its application in catalytic processes, particularly in redox reactions. Catalytic functionality arises from the ability of ceria to generate and annihilate oxygen vacancies under reaction conditions. The oxygen atoms released from the lattice during catalytic reactions leave behind vacancies that act as active sites for subsequent reactions. These vacancies, dynamically forming and dissolving at the surface, enable the efficient interaction with reactants and intermediates. Surface vacancies are especially critical, as they facilitate the adsorption and activation of reactants, driving heterogeneous catalysis. A nuanced understanding of oxygen vacancies also requires attention to their charge states and associated polarons, which can trap electrons within the lattice. These electronic interactions are crucial for describing ceria’s defect chemistry. The interplay between charge carriers and lattice distortions profoundly impacts its electromechanical properties, including electrostriction. Defect formation in ceria involves the migration of oxygen ions to interstitial positions, leaving behind doubly charged vacancies in the original lattice sites. Using Kröger–Vink notation [24], these processes can be modeled to describe the material’s defect behavior. During catalytic oxidation reactions, lattice oxygen is released as molecular oxygen, creating reactive oxygen species on the surface. These reactive species enhance the catalytic efficiency of ceria, making it highly effective in oxidation and reduction reactions.

## 3. Synthesis Approach and Structural Features

Rare-earth high-entropy oxides (RE-HEOs) combine the structural stability of the crystal system with a unique configurational entropy-driven phase stabilization inside the crystal lattice. The synthesis methods, compositional variations, and resulting morphologies are central to understanding and optimizing the properties of RE-HEOs for advanced applications. Various synthesis techniques have been employed to obtain phase-pure RE-HEOs, each influencing the resulting unique structural and surface characteristics. The solid-state reaction method represents one of the earliest and simplest approaches for producing RE-HEOs. This method typically involves the high-temperature calcination of equimolar metal oxides or salts, resulting in single-phase bulk materials. Rost et al. [2] initially demonstrated the feasibility of this synthesis to produce rock-salt-type RE-HEOs, a finding later corroborated by Xue et al. [38], who synthesized (La_0.2_Nd_0.2_Sm_0.2_Eu_0.2_Gd_0.2_)_2_Ce_2_O_7_. While the solid-state reaction method is effective for achieving a uniform elemental distribution and excellent thermal stability, it often requires high calcination temperatures, limiting its capacity to produce nanoscale morphologies with higher surface areas. These limitations served as a primer for the development of solution-based synthesis methods.

The sol–gel method has proven particularly effective in producing RE-HEOs with relatively high surface areas and homogeneous particle distributions. In the study by Tatar et al. [39], a citrate-based sol–gel synthesis was employed to prepare several phase-pure ceria–zirconia-based RE-HEOs, achieving a single-phase cubic fluorite structure with enhanced oxygen vacancy concentrations. The sol–gel process offers advantages such as the use of lower synthesis temperatures and the ability to tailor porosity by adjusting the gelation parameters. Modified versions of this method, incorporating citric acid as a chelating agent, further improve compositional uniformity and thermal stability. The gelation process, followed by calcination at controlled temperatures, resulted in a flake-like morphology interspersed with hollow tube-like structures. These morphological features enhanced the material’s surface area and porosity.

Solution combustion synthesis represents another innovative approach [40], characterized by rapid exothermic reactions between metal precursors and fuels. This method has been widely utilized to produce highly porous, crystalline RE-HEOs at relatively low temperatures [41].

Spray pyrolysis (SP) and nebulized spray pyrolysis (NSP) are advanced methods capable of producing nanostructured RE-HEOs with controlled morphologies [42]. Sarkar et al. utilized NSP to synthesize rock-salt-type RE-HEOs, achieving sub-micron spherical particles with uniform elemental distribution. These methods stabilize metastable phases through rapid quenching, resulting in materials with high catalytic stability and specific surface areas, ideal for industrial-scale applications.

The sol–gel and solution combustion methods excel in producing materials with high surface areas and porous networks. The ability to manipulate defect chemistry, particularly oxygen vacancies, further enhances the functional properties of RE-HEOs. These vacancies act as active sites for catalytic reactions and are pivotal for applications like CO oxidation and CO_2_ hydrogenation. The configurational entropy inherent in RE-HEOs stabilizes these defect structures, ensuring consistent performance under extreme conditions.

**Figure 1:**
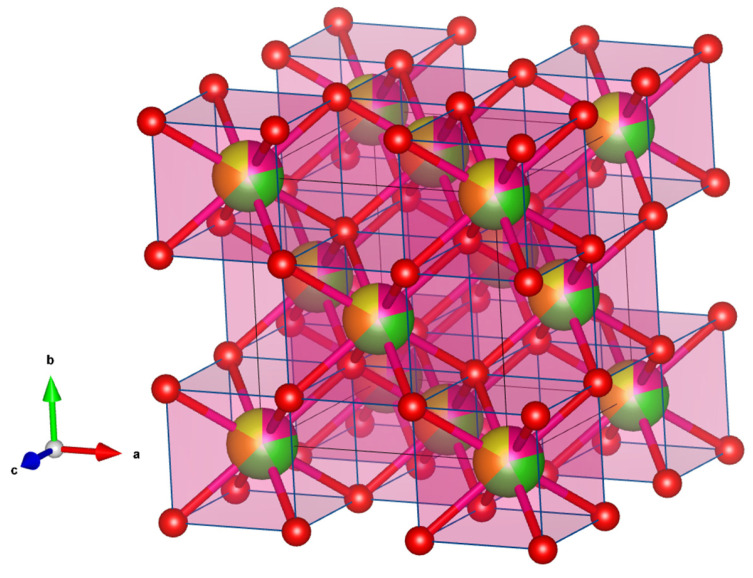
Fluorite-type crystal structure of ceria-based high-entropy oxides showing the incorporation of 5 cations with similar ionic radius and oxidation state and the same coordination number into a single crystallographic position, with each cation occupying 1/5 of the position (each cation depicted in different color), while the anion remains untouched. The a, b, and c axes in the image represent the crystallographic orientation of the unit cell, as they define the three-dimensional lattice directions in the crystal structure.

The structural stability of RE-HEOs is attributed to their ability to form solid solutions where cations occupy specific lattice sites, resulting in a uniform elemental distribution and high configurational entropy. These materials are typically stabilized in fluorite, perovskite, and pyrochlore structures, depending on the cationic combination and synthesis approach. For instance, fluorite-type RE-HEOs such as Ce_0.2_Zr_0.2_La_0.2_Pr_0.2_Y_0.2_O_2_ [39,43], synthesized through sol–gel methods, adopt a cubic symmetry (*Fm*-3*m* space group), with Ce^3+^/Ce^4+^ ions occupying the 4*a* positions, and oxygen anions filling the tetrahedral position, as shown in Figure 1. This arrangement promotes the formation of oxygen vacancies. Pyrochlore-structured RE-HEOs exhibit an *FCC* lattice where rare-earth cations occupy the A-site, and transition metals stabilize the B-site. These structures are particularly notable for their thermal stability and defect engineering capabilities. Variations in the ionic radius and oxidation state among the constituent cations lead to lattice distortions, which further enhance oxygen mobility and catalytic activity. For example, in Nd_2_Zr_2_O_7_, the introduction of oxygen vacancies through cation substitution has been shown to significantly improve ionic conductivity [44]. In perovskite-type structures, cations are distributed across the A- and B-sites, creating a versatile platform for tailoring electronic and catalytic properties [44,45]. These materials have demonstrated exceptional performance in electrocatalytic oxygen reduction reactions [45] and hydrogen evolution reactions [46], with enhanced activity attributed to the synergistic effects of rare-earth and transition metal cations. The multicomponent composition of these RE-HEOs ensures thermal stability and resistance to phase degradation under harsh reaction conditions. The compositional flexibility of RE-HEOs enables the fine-tuning of properties for specific applications. The inclusion of cerium in fluorite structures facilitates the redox cycling between Ce^3+^ and Ce^4+^, creating a high density of active sites for catalytic reactions. Additionally, the incorporation of smaller cations like zirconium enhances lattice contraction, which increases the material’s stability at higher temperatures [22]. Advanced characterization techniques such as X-ray diffraction (XRD), Raman spectroscopy, and transmission electron microscopy (TEM) are typically used to analyze the structural integrity and defect chemistry of RE-HEOs.

## 4. Next-Generation Technologies

As the demand for cleaner energy, faster computation, and more efficient resource utilization grows, next-generation technologies provide the innovative solutions required to address these needs. For instance, renewable energy technologies like solar cells [47] and hydrogen fuel cells [48] are considered to be the perfect “weapon” to fight the rapid climate change by reducing reliance on fossil fuels. Similarly, materials science breakthroughs, such as high-entropy oxides and, further, rare-earth high-entropy oxides, offer the development of multifunctional systems that integrate durability, efficiency, and sustainability. These technologies are beneficial not only for addressing environmental and social challenges but also for ensuring global competitiveness in this rapidly advancing technological field. In the field of energy technologies, RE-HEOs were successfully incorporated in solid oxide fuel cells (SOFCs) [49,50,51,52], due to their ionic conductivity and thermal stability. Materials such as Gd-doped CeO_2_ have notable performance as electrolytes, allowing for efficient ion transport and reducing the operational temperatures in SOFCs [53,54]. This reduction in temperature enhances system longevity and lowers the overall costs, making SOFCs more attractive for future usage. Furthermore, RE-HEOs are successfully integrated into thermochemical water-splitting cycles for hydrogen production [55]. The presence of oxygen vacancies, which can be tailored through rare-earth doping, significantly facilitates the redox reactions necessary for splitting water molecules. This process (especially when driven by solar energy) represents a sustainable pathway for generating hydrogen. RE-HEOs with perovskite or fluorite structures offer high capacity and cyclic stability, which potentially makes them suitable for next-generation energy storage devices [56,57]. Moreover, the thermal and chemical robustness provided by high-entropy material design could ensure a consistent performance in demanding operational environments, such as electric vehicles and grid-scale energy storage systems [58,59]. The catalytic properties of RE-HEOs are another aspect of significant impact, particularly in environmental and industrial applications. The presence of rare-earth elements in catalysts enhances the catalytic efficiency of materials, primarily through the ability of these elements to create oxygen vacancies [22]. These vacancies serve as active sites for catalytic reactions, enabling the efficient oxidation of pollutants and the reduction of greenhouse gases. In electronics and optoelectronics, the rare earths present in high-entropy oxides could contribute to the development of devices with enhanced performance and functionality [60]. The dielectric properties of HEOs, influenced by the electronic structure of rare-earth elements, have also been investigated [61]. Their high permittivity and low dielectric loss make them suitable for miniaturized electronic components in portable and high-speed devices. Additionally, HEOs with tunable band gaps are being explored for photovoltaic applications, where their ability to absorb and convert sunlight into electricity is optimized by a precise compositional control [62]. Only several rare-earth metals in HEO systems are used in electronic applications due to the challenges posed by complex structures in multicationic systems, as it has still not been quite investigated how each element may contribute to the cocktail effect [62]. Another domain where high-entropy oxides with rare-earth metals are investigated is magnetic and spintronic technologies [63,64,65]. Rare-earth elements add unique magnetic properties to high-entropy systems, enabling the design of advanced materials for data storage, magnetic sensors, and spintronic devices. In general, the integration of rare-earth elements into high-entropy oxide systems enables the customization of materials for specific applications. This multifunctional nature of high-entropy oxides and rare-earth-based high-entropy oxides makes them suitable materials for future applications.

### 4.1. Energy Conversion and Storage

#### 4.1.1. Application in Solid Oxide Fuel Cells

**Figure 2:**
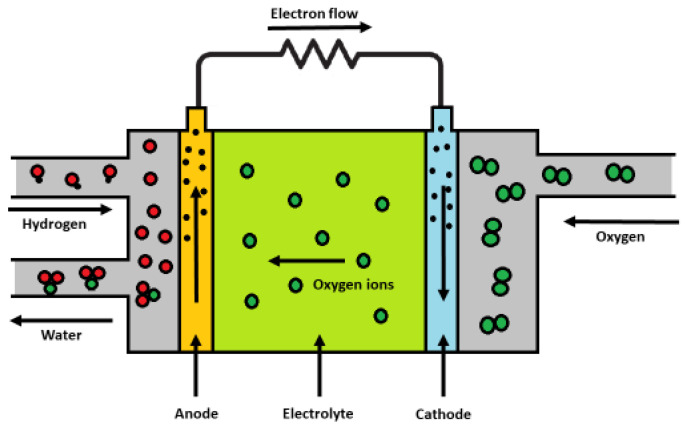
General scheme of a solid oxide fuel cell (SOFC).

In the past few decades, the need for renewable energy sources has become more and more emphasized. Therefore, research activities have headed the way to develop green technologies to address the existing environmental challenges. Figure 2 shows a scheme of a solid oxide fuel cell. Here, two chemical reactions take place, i.e., the reduction of oxygen (from air) into oxygen anions (Equation (1), cathode) and the oxidation of hydrogen with oxygen anions to form water (Equation (2), anode). In other words, SOFCs use the tendency of oxygen and hydrogen to react. Thus, this reaction transforms the chemical energy in fuels (natural gas, biofuel, etc.) to electrical energy, with no emission of harmful gases [56,57,66,67]

(1) $$C:O_{2}+2e^{-}\to 2O^{2-}$$

(2) $$A:H_{2}+O^{2-}\to H_{2}O+2e^{-}$$

Yttrium- and scandium-stabilized zirconia (YSZ and ScSZ) and RE-doped ceria are among the most common ion conductor electrolytes in SOFCs because of their chemical stability and excellent ionic conductivity [68,69].

One of the most notable roles of RE-HEOs in SOFCs lies in their development as advanced electrolytes. Traditional materials, such as yttrium-stabilized zirconia and gadolinium-doped ceria [70], are effective but encounter limitations in medium-to-low-temperature operations [71,72,73] and long-term aging. HEOs have addressed these limitations by incorporating multiple elements, alongside rare-earth elements, to create highly stable and conductive oxide ion pathways [74,75]. These materials demonstrate significant ionic conductivity, surpassing conventional electrolytes, while maintaining excellent chemical and thermal stability. The configurational entropy inherent in RE-HEOs reduces phase separation, ensuring durability under harsh operating conditions [76]. For instance, the synthesis of high-entropy oxides such as Ce-doped systems (Ce_1–x_(Gd_1/5_Sm_1/5_Er_1/5_Y_1/5_Bi_1/5_)_x_O_2–δ_) [77] has resulted in electrolytes with exceptional ionic conductivity and reduced activation energies for oxygen ion transport [77,78]. These advancements are particularly valuable for operating at reduced temperatures, which enhances the efficiency of SOFCs. HEOs are also often used as cathode materials in SOFCs [76,79,80]. Xu et al. [81] also reported the importance of Ruddlesden–Popper perovskites as promising electrocatalysts for O_2_ reduction/evolution and H_2_ evolution for SOFCs. By incorporating rare-earth and transition metals into the simple perovskite lattice, cathode and anode materials with mixed electronic ionic conductivities have been produced. Ruddlesden–Popper perovskites showed electrocatalytic performance that even surpassed that of simple perovskites due to their unique physicochemical properties and layered structure.

The application of rare-earth elements as fuel electrodes in SOFCs has also gained traction due to their remarkable catalytic properties and resilience to degradation. Nickel-based electrodes, though widely used, face challenges such as carbon deposition and sulfur poisoning during operation with hydrocarbon fuels [82,83]. The inclusion of rare-earth elements such as La, Nd, Sm, and Gd significantly contributes to the stabilization of oxygen vacancies, crucial for maintaining a high ionic conductivity under SOFC operating conditions; this method has been employed to fine-tune the properties of RE-HEOs. Materials such as (La_0.2_Nd_0.2_Sm_0.2_Eu_0.2_Gd_0.2_)_2_Zr_2_O_7_ demonstrated increased thermal stability and high ionic conductivity, which makes them suitable as electrolytes in SOFCs [49]. By fine-tuning the entropy effects and their elemental compositions, these materials enhance the compatibility of electrodes with electrolytes and interconnects. For instance, compositions like (La_0.2_Pr_0.2_Nd_0.2_Sm_0.2_Gd_0.2_)_2_CuO_4_ exhibit a high degree of stability and improved conductivity and serve for chromium-resistant air electrodes [50]. Their high-entropy design inhibits Ba polarization, thereby enhancing stability and reducing Cr poisoning effects during prolonged operation. This demonstrates their capability to withstand harsh SOFC environments while maintaining high catalytic activity for the oxygen reduction reaction. This directly proves that rare-earth-containing high-entropy oxides enable efficient electrochemical reactions by stabilizing oxygen vacancies and facilitating the adsorption and dissociation of molecules. This capability is critical for hydrocarbon-based SOFCs [52,84,85,86,87], allowing for the direct utilization of fuels such as methane and ethanol without pre-reforming, thereby reducing system complexity and costs. In the context of air electrodes, RE-HEOs have proven instrumental in optimizing oxygen reduction reaction and oxygen evolution reaction kinetics [88]. Their high configurational entropy contributes to excellent mixed ionic–electronic conductivity, crucial for expanding the reaction zones in porous electrodes [82,87,88]. For instance, high-entropy perovskites with rare-earth elements have demonstrated exceptional ORR activity due to their flexible lattice structures, which accommodate multiple valence states and facilitate an efficient oxygen ion transport. This is specifically crucial in compositions like (Pr_0.2_Sm_0.2_Nd_0.2_Gd_0.2_La_0.2_)BaCo_2_O_5+δ_, which enabled operation for over 142 h at 800 °C, with low degradation rates [51]. This stability translates into prolonged cell lifespans and enhanced performance metrics, including higher power densities and lower polarization losses.

#### 4.1.2. Achievements in Hydrogen Production

Hydrogen as an energy source is a clean, eco-friendly, and the most promising alternative to fossil fuels. This is why, in the past few years, research has focused on finding materials that could enhance electrochemical water splitting [89,90,91,92]. Sun et al. [93] showed that introducing and discovering new unusual active sites contributes reaction pathways with lower activation barriers, helping with reactions that are restrained by high-energy conditions. Electrochemical water splitting appears as a favorable technology for green hydrogen production using materials that include transition metals, rare-earth metals, and noble metals. During electrocatalysis, two half-reactions take place on the cathode and the anode (Equations (3) and (4)):

(3) $$K:2H^{+}(aq)+2e^{-}\to H_{2}(g)$$

(4) $$A:2H_{2}O\left(l\right)\to 4H^{+}+4e^{-}+O_{2}(g)$$

**Figure 3:**
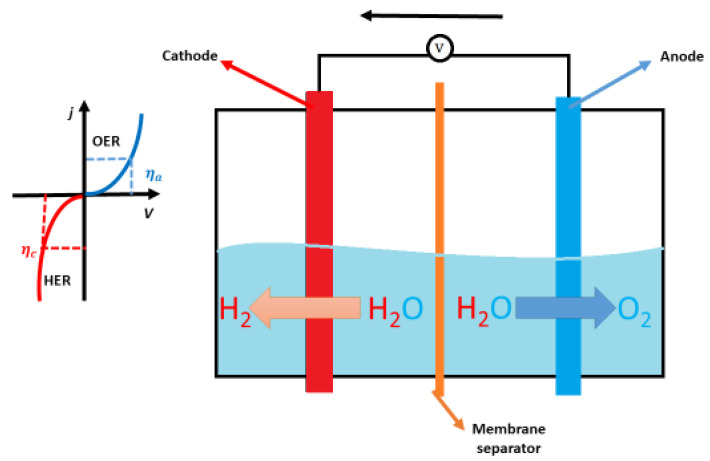
Schematic representation of the electrochemical water splitting reaction.

However, the energy barrier needs to be overcome for an electrochemical reaction to occur (overpotential, *η*). This is where catalysts are needed, because they should minimize the overpotential on the cathode (*η*_c_) and anode (*η*_a_). The electrochemical water splitting reaction is visualized in Figure 3.

**Figure 4:**
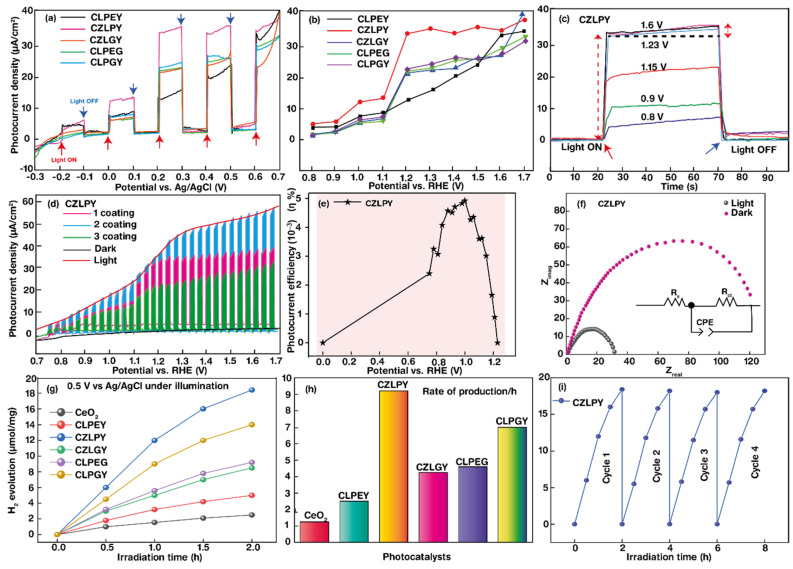
Photoelectrochemical (PEC) performance of CLPEY, CZLPY, CZLGY, CLPEG, and CLPGY was evaluated using LSV (**a**,**b**,**d**) under lights on (red arrow) and lights off (blue arrow) (**a**), photoswitching analysis (**c**), coating thickness-dependent LSV (**d**), ABPE efficiency plots (**e**), EIS (**f**), hydrogen evolution measurements (**g**,**h**), and stability testing (**i**), as reported by Nundy et al. [43].

The elemental compositions of RE-HEOs are carefully engineered to optimize their catalytic performance. Most of the rare-earth HEOs have been used for oxygen evolution reactions (OERs) because of their ability to oxidize other materials due to their high OSC [94,95,96]. However, compositions such as Ce_0.2_Zr_0.2_La_0.2_Pr_0.2_Y_0.2_O_2_ (CZLPY) have been synthesized, along with Ce_0.2_La_0.2_Pr_0.2_Eu_0.2_Y_0.2_O_2_ (CLPEY), Ce_0.2_Zr_0.2_La_0.2_Gd_0.2_Y_0.2_O_2_ (CZLGY), Ce_0.2_La_0.2_Pr_0.2_Eu_0.2_Gd_0.2_O_2_ (CLPEG), and Ce_0.2_La_0.2_Pr_0.2_Gd_0.2_Y_0.2_O_2_ (CLPGY), as shown in Figure 4, to investigate the synergistic effects of elements in hydrogen evolution reactions [43]. Each of these elements contributes distinct properties: cerium provides redox flexibility, enabling oxygen vacancy formation; zirconium enhances thermal stability; lanthanum promotes lattice homogeneity; praseodymium adds redox versatility due to its mixed oxidation states; and yttrium stabilizes the fluorite lattice. These multicomponent oxides exhibit a bandgap between 1.9 and 3.0 eV, suitable for visible-light photocatalysis, and also for UV light catalysis. Bandgap tuning, facilitated by the incorporation of rare-earth and transition metals, is critical for maximizing light absorption and optimizing the charge carrier dynamics essential for hydrogen production. In this regard, the PEC performance of five RE-HEOs was evaluated under chopped dark and solar light conditions. Linear sweep voltammetry was recorded against both Ag/AgCl (V) and RHE (V) references. The photoswitching behavior of Ce_0.2_Zr_0.2_La_0.2_Pr_0.2_Y_0.2_O_2_ was assessed at multiple potentials (0.8–1.6 V vs. RHE). Coating thickness variations (1, 2, 3 layers) were analyzed via LSV under light/dark cycles. Applied-bias photon-to-current efficiency (ABPE, *η*%) was plotted against the RHE potential. Electrochemical impedance spectroscopy (EIS) was performed in dark and light conditions. Hydrogen evolution was monitored for CeO_2_ and all electrode variants, with production rates quantified after 2 h of irradiation. Finally, the stability of Ce_0.2_Zr_0.2_La_0.2_Pr_0.2_Y_0.2_O_2_ was examined over four catalytic cycles to assess its long-term activity. The results for these materials indicated a significant enhancement in photocatalytic activity compared to that of the “parent” oxides (pure CeO_2_), achieving hydrogen production rates up to 9.2 µmol/mg/h, while pure cerium oxide achieved 0.8 µmol/mg/hour under the same conditions, as shown in Figure 4. Computational and experimental studies demonstrated that these defects increase the adsorption and activation of water molecules, leading to improved catalytic kinetics [43]. Further, the role of platinum and other noble metals in modifying HEOs has been explored to amplify HEO catalytic capabilities [44,97,98]. Platinum-modified HEOs, synthesized by decorating oxide surfaces with platinum nanoparticles, have shown excellent HER performance across a universal pH range [44]. The interaction between platinum and oxygen vacancies on the RE-HEO surface enhances the catalytic efficiency by optimizing hydrogen adsorption and desorption dynamics.

### 4.2. Catalysis and Environmental Achievements

#### 4.2.1. CO Oxidation

Carbon monoxide (CO) is a colorless, flammable gas that is also very toxic in high concentration in closed space [99]. The main source of carbon monoxide is the incomplete oxidation of fuel systems such as oil, natural gas, coal, wood, etc. [100]. Considerable amounts of CO are also detected in major urban areas due to emissions from the exhaust of internal combustion engines, fuel-burning stoves, and domestic heating, affecting environmental and climate processes [101]. The most representative description of CO oxidation is a simple reaction (Equation (5))

(5) $$2CO\left(g\right)+O_{2}(g)\to {2CO}_{2}(g)$$

To minimize the concentration of carbon monoxide in the atmosphere and to protect the environment, CO oxidation reactions in the field of heterogeneous catalysis have been studied. To reduce and convert CO in the atmosphere, early research focused on using ozone and catalysts such as simple metal oxides or Ag, Au, Pt, etc. The main goal was to obtain the reaction product CO_2_, as it was believed to be inert and unable to interfere with the reaction as long as no reducing species were adsorbed. CO oxidation can also be seen in electrochemistry when carbon monoxide is dissolved in water, using the hydroxyl (OH) group as an oxidant. To clean hydrogen streams, in this case, to eliminate CO by oxidating it to CO_2_, gold nanoparticles can be used even below room temperature. The catalyst surface is also a viable factor as regards the CO oxidation reaction. A higher specific surface area allows more CO molecules to be adsorbed onto the catalyst material and therefore react with oxygen, transforming to carbon dioxide and, at the same time, oxidizing metallic sites and thus converting the material to an oxide phase. Factors that can also affect the CO oxidation reaction are catalysts’ chemical structure, crystallite size, temperature, weight, and water vapor [102,103].

**Figure 5:**
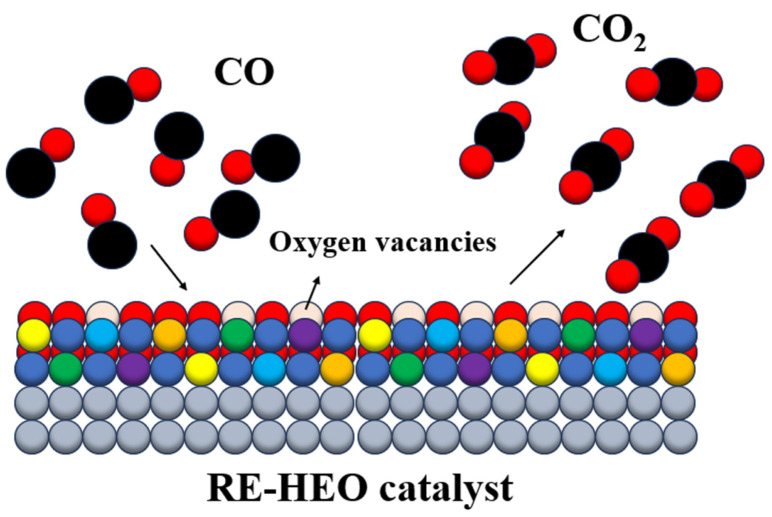
Schematic overview of CO oxidation catalysis over the oxygen vacancies formed on the surface of RE-HEOs.

Two main categories exist when talking about catalysts for CO oxidation: supported noble metal catalysts and non-noble metal catalysts. For non-noble metal catalysts, transition metals are primarily used to synthesize oxides such as Fe-, Mn-, Co-, Cr-, and Cu-based oxides, and also rare-earth elements such as Ce, Zr, La, Nd, Sm, Pr, etc. Their abundant surface-activated oxygen enables them to keep the CO oxidation functionality under cycling conditions [104]. In recent years, several materials with extraordinary potential as CO oxidation catalysts have been reported. Their elemental compositions typically include rare-earth cations often paired with transition metals like chromium, molybdenum, and tungsten. These compositions are designed to exploit the redox-active nature of transition metals and the structural stability provided by rare-earth elements. The catalytic performance of high-entropy rare-earth perovskite nanofibers in carbon monoxide oxidation was studied by Krawczyk et al. [105]. Using the glycothermal method, they managed to synthesize the rare-earth high-entropy perovskite oxide (Y_0.2_La_0.2_Nd_0.2_Sm_0.2_Gd_0.2_)CoO_3_, which demonstrated exceptional catalytic performance due to its balanced incorporation of these elements. The presence of rare-earth cations contributes to maintaining the integrity of the crystalline structure at high temperatures, while the transition metals enhance oxygen vacancy formation. These vacancies serve as active sites where CO molecules can adsorb and react with lattice oxygen to form CO_2_ (Figure 5).

**Figure 6:**
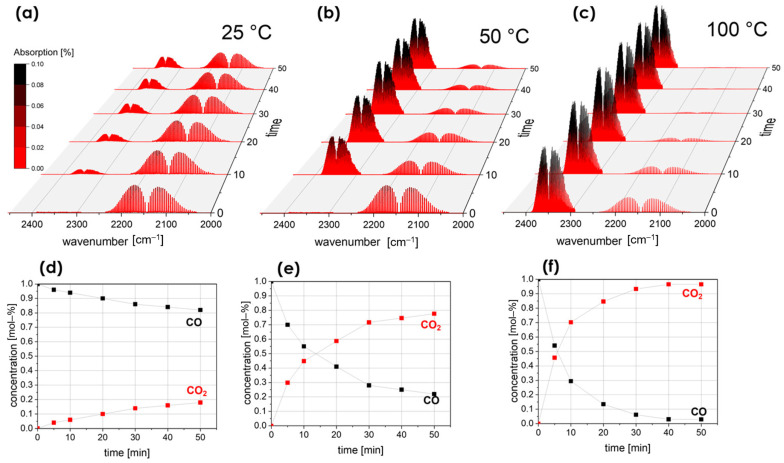
FTIR spectra of the gas phase over RECO GT 300 °C (RECO, RE = Y, La, Nd, Gd, Sm, C=Co, O=O_3_); GT, synthesis temperature) at (**a**) 25 °C, (**b**) 50 °C, and (**c**) 100 °C (0–50 min). CO and CO_2_ concentrations from FTIR analysis over time at (**d**) 25 °C, (**e**) 50 °C, and (**f**) 100 °C, as reported by Krawczyk et al. [105].

The synthesis path consisted of mixing precursor salts with a solvent mixture of 1,4-butanediol and diethylene glycol in a volume ratio of 9:1, heating at 250 °C for 72 h, removing organic by-products by high-speed centrifugation, and drying the product in a vacuum oven at 120 °C. A custom-designed method was implemented to evaluate the catalytic efficiency of the synthesized fibrous materials in oxidizing carbon monoxide (CO) to carbon dioxide. Figure 6 presents the FTIR spectra recorded over 50 min at temperatures ranging from 25 °C to 100 °C, illustrating the gradual formation of CO_2_, along with the CO oxidation behavior that showed conversion of 78% at 50 °C and of 97% at 100 °C, which are quite effective results in the low-temperature and mild conditions applied. This study showed that by incorporating rare-earth elements in the high-entropy composition, the synergistic effect occurs, which enhances the catalytic properties of high-entropy oxide catalysts [105].

For example, studies on fluorite-structured RE-HEOs, such as (Ce_0.2_La_0.2_Sm_0.2_Gd_0.2_Y_0.2_)O_2_, have shown that their high oxygen vacancy concentrations facilitate a rapid CO conversion, achieving nearly complete oxidation at temperatures as low as 100 °C. Modifying the Ce-based fluorite phase by adding cationic elements like La, Nd, Pr, Sm, Y, Zr, Fe, and Al, enhances the thermal stability, redox properties, and oxidation activity of HEOs in general. The RE-HEO with the formula (CeLaPrSmY)O_2-y_ was synthesized using the sol–gel method in which the composition of cerium varied from 20 to 80 at. %, while equimolar concentrations of other rare-earth elements were maintained. Metal precursors were mixed within the aqueous solution and bonded with a complexing agent, in this case, polyvinylpyrrolidone. The mixture was stirred and dried to form a hard gel at 110 °C, which was subjected to a box furnace for 2 h at 500 °C to obtain the RE-HEO. Adding more rare-earth elements that had different ionic radii compared to Ce^4+^, disrupted the ceria lattice, which as a consequence, destabilized the lattice oxygen, allowing it to react more rapidly with carbon monoxide. The study showed that the activity of HEO in CO oxidation immensely improved by increasing the cerium content to 80% and adding rare-earth elements like La, Nd, Zr, and Pr onto the cerium site, compared to the activity measured when using pure CeO_2_ [106].

Reducing the crystallite size positively affects CO oxidation till a certain size limit, after which it decreases proportionally. Melting point depression at the nanoscale can take place on the particles and increase the flexibility of metal atoms over the support surface. For example, Au has always been seen as an inert metal for catalysis until Haruta et al. [107] managed to decrease the crystallite size to less than 10 nm to oxidize CO. The oxidation was successful, but on the other hand, the catalyst suffered problems such as deactivation in indoor light and storage, upscale value, etc. Temperature is one of the main factors for the deactivation of catalysts, as it influences the degree of crystallization, the crystallite size, the formed metal oxide, and the surface area of catalysts. Two main temperatures need to be taken in hand when talking about the effect of temperature on the CO oxidation reaction, which are the calcination temperature and the catalytic reaction temperature. Using higher calcination temperatures, phases with a high degree of crystallization can form, thus providing a higher % of CO oxidation than those obtained at lower temperatures. It also affects the formed metal oxide, the support phase, and the oxidation state. As reported by Chang et al. [108], calcinating CeO_2_ at 400 °C instead of 200 °C increased CO oxidation by more than 20%, which can be comparable to the result obtained by increasing the concentration of Ce^4+^ species by 5%. The catalytic reaction temperature also greatly affects the CO conversion %, as high temperatures can lead to catalyst sintering. Halim et al. reported that when using high reaction temperatures such as 400 °C and 500 °C for the oxidation of CO, the catalytic activity increased up to 400 °C, reached its equilibrium, and then decreased up to 500 °C due to the sintering effect [109]. Overheating of the active sites of the catalysts can also occur prior to CO oxidation due to the release of excessive heat from the exothermic oxidation of carbon monoxide to carbon dioxide. Enlarging the catalyst weight proportionally increases its total surface area and the number of sites available for the reaction of the catalyst, which as a consequence, increases the conversion % of CO to CO_2_. Soliman reported that increasing the weight of a metal oxide, consisting of Ce, Cu, Y, Fe, and Al, from 0.3 g to 3 g raised the conversion % from 48% to 80% [102].

Transition metal-doped RE-HEOs, such as those incorporating Cr, further enhance the reaction kinetics by lowering the activation energy required for oxygen exchange. The balance between the redox activity of transition metals and the structural stability provided by rare-earth elements ensures a sustained catalytic performance even under harsh operating conditions [106]. Another significant achievement in the development of RE-HEOs for CO oxidation is their inherent resistance to thermal degradation. Conventional catalysts often suffer from sintering and phase segregation at elevated temperatures, leading to a loss of activity. In contrast, RE-HEOs maintain their single-phase structure and catalytic properties due to the stabilizing effect of configurational entropy. This thermal stability has been demonstrated in materials that retain high activity after prolonged exposure to temperatures exceeding 800 °C. Such resilience makes RE-HEOs ideal for applications in automotive exhaust systems and industrial processes that involve high-temperature oxidation reactions [106].

#### 4.2.2. CO_2_ Reduction

In recent years, CO_2_ has become the main topic when talking about global warming and environmental control. It belongs to the group of greenhouse gases that contribute to global warming by gripping infrared radiation and holding it back from going into space. CO_2_ emission contributes over 60% to global warming, since fossil fuels are still the main sources of energy. To overcome that, several technologies have been proposed to capture and use CO_2_ cost-effectively and reasonably, such as conversion to chemicals, reduction, welding, water treatment, hydrogenation, etc. [110,111].

The utilization of CO_2_ as a raw material for producing valuable and usable compounds such as carbon monoxide, formic acid, formaldehyde, methane, methanol, etc., is an inviting way to make carbon flow in the ecosystem. Various methods have been investigated for CO_2_ conversion, but the main approaches involve photocatalysis, thermal catalysis, and electrocatalysis. High CO_2_ reduction is achieved in thermal catalysis, but conditions of high pressure and high temperature lead to significant energy cost and safety issues. When talking about electrocatalysis, an external electric field is used, in which there is a compromise between selectivity and activity, as a result of overpotential. An encouraging way for artificial carbon recycling to address the global challenges in preserving energy is developing electrocatalysts that have the potential to selectively reduce CO_2_ to oxygenated hydrocarbon products. Raciti and Wang [112] reported Cu electrocatalysts for CO_2_ reduction, where it was seen that structural effects, involving surface crystalline facets and grain boundaries, morphology, porosity, and pH, had a huge impact on the conversion rate of CO_2_. The most promising way of CO_2_ reduction lies in photocatalysis, which uses solar energy to convert CO_2_ into organic compounds that can be used for further chemical reactions. It depends on solar energy for CO_2_ reduction, shows small energy consumption, requires reasonable operation conditions, and achieves an enormous conversion % in CO_2_ reduction [113].

Recent advances in RE-HEOs development have expanded our understanding of their compositional frameworks, synthesis techniques, reaction mechanisms, and catalytic efficiencies. One of the most promising advances in RE-HEOs for CO_2_ hydrogenation is the development of fluorite-structured systems. A notable example is (Ce_0.2_Zr_0.2_La_0.2_Nd_0.2_Sm_0.2_)O_2−δ_, which demonstrated significant catalytic performance for CO_2_ conversion under photocatalytic conditions [22].

**Figure 7:**
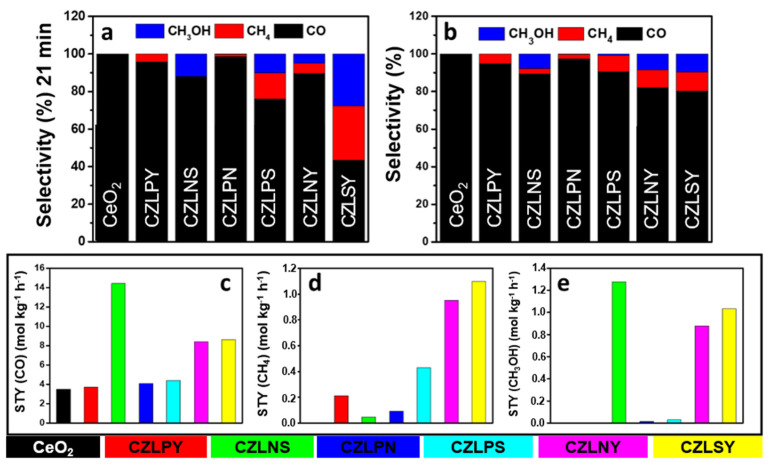
RE-HEO selectivity toward reaction products at (**a**) 21 min and (**b**) 189 min. Space–time yield for HEO catalysts of reaction products (**c**–**e**), as reported by Tatar et al. [22].

This material achieved a remarkable production of 14.4 mol CO kg^−1^ h^−1^ and 1.27 mol CH_3_OH kg^−1^ h^−1^, with a high selectivity of 89.26% for CO and 7.84% for methanol, as shown in Figure 7. Along Ce_0.2_Zr_0.2_La_0.2_Nd_0.2_Sm_0.2_)O_2−δ_ (CZLNS), five more RE-HEOs were investigated—Ce_0.2_Zr_0.2_La_0.2_Pr_0.2_Y_0.2_O_2−δ_ (CZLPY), Ce_0.2_Zr_0.2_La_0.2_Pr_0.2_Nd_0.2_O_2−δ_ (CZLPN), Ce_0.2_Zr_0.2_La_0.2_Pr_0.2_Sm_0.2_O_2−δ_ (CZLPS), Ce_0.2_Zr_0.2_La_0.2_Nd_0.2_Y_0.2_O_2−δ_ (CZLNY), and Ce_0.2_Zr_0.2_La_0.2_Sm_0.2_Y_0.2_O_2−δ_ (CZLSY)—shown in Figure 7. Advanced characterization techniques, such as X-ray photoelectron spectroscopy and in situ diffuse reflectance infrared Fourier transform spectroscopy, proved the formate-driven mechanism underlying CO_2_ activation in these systems. Fluorite RE-HEOs, such as Ce_0.5_(LaPrSmY)_0.5_O_2−y_, are also used as catalysts for the synthesis of dimethyl carbonate from carbon dioxide and methanol [114]. In a study, the catalyst was synthesized using the anchoring method, which included the adsorption of rare-earth metal cations by negative functional groups on the surface of graphene oxide. By dissolving graphene oxide in ethylene glycol and mixing it with a certain molar ratio of rare-earth nitrates, refluxing for 4 h at 170 °C, and calcinating at temperatures over 500 °C, high-entropy rare-earth oxides were obtained. The direct synthesis of DMC from CO_2_ and CH_3_OH benefited from the catalytic performance of Ce_0.5_(LaPrSmY)_0.5_O_2−y_, showing a yield of 7.2 mmol/g using 0.05 g of catalyst [115]. The RE-HEO was much faster (almost 2 times) in converting DMC to the desired products compared to pristine CeO_2_, due to a higher specific surface area, larger pores, and gaps between the layers that provided more active sites. Adding more rare-earth metals onto the same lattice site with different atomic radii and bond lengths led to severe distortion of the lattice, which increased the number and size of the oxygen vacancies and promoted the electron transfer of active species [115]. The catalytic performance of RE-HEOs for CO_2_ hydrogenation is linked to their defect chemistry, particularly the formation and stabilization of oxygen vacancies. For example, the introduction of transition metals such as Ni or Cu into rare-earth element materials showed significant enhancement of the reactivity and selectivity of these materials [116].

### 4.3. Emerging Applications Beyond Energy and Environment

One of the most promising next-generation technology applications is quantum materials and spintronics, where the material’s ability to sustain long-range magnetic interactions and modulate electron correlation effects has directly influences on high-performance memory storage, magneto-resistive devices, and topological insulators [117,118]. The role of transition metals within the high-entropy lattice induces tunable exchange interactions, offering a robust platform for multifunctional magnetic and electronic materials [119]. In aerospace and defense technological applications, HEOs exhibit superior phase stability under extreme thermal and mechanical stress [120]. Their configurational entropy minimizes atomic diffusion and grain growth at elevated temperatures, making them ideal candidates for ultra-high-temperature ceramics, thermal barrier coatings, and radiation-resistant materials [2]. These attributes position HEOs as potential replacements for conventional oxides in hypersonic vehicles, space propulsion systems, and nuclear reactor components [13]. The biomedical applications of HEOs have also gained attention, particularly in bioelectronics, antibacterial coatings, and implantable materials [56]. The incorporation of biocompatible elements such as cerium, yttrium, and calcium enhances their functional integration with biological systems [24]. Rare-earth high-entropy oxides are a relatively new class of materials that have emerged as a promising extension of high-entropy materials. Initially, RE-HEOs were mostly investigated for catalytic applications, particularly in oxygen evolution reactions, hydrogen evolution reactions, CO oxidation, and CO_2_ reduction, where their multi-element synergies enhance surface activity and stability under harsh conditions [121]. However, recent research demonstrated their multifunctionality, extending their applications beyond catalysis into non-conventional fields such as optoelectronics, photoluminescent material science, and thermal barrier coating production. Recent advancements show that RE-HEOs are adaptable to optoelectronic applications due to their bandgap tunability and photoluminescence properties. For example, Kumbhakar et al. [122] investigated high-entropy fluorite oxides and demonstrated that these materials offer a tunable bandgap range of 2.0–3.5 eV, which makes them ideal candidates for photovoltaic and LED applications. Similarly, Nundy et al. [43] validated the photoluminescent potential of RE-HEOs, showing that La-, Ce-, Pr-, Eu-, Gd-, Y-, and Zr-based RE-HEOs have stable light emission properties, which could be interesting for display technologies and semiconductor applications. Optoelectronics is an interdisciplinary field integrating photonics and electronics to manipulate and control light for applications ranging from telecommunications and solid-state lighting to advanced sensing technologies and quantum information processing [123]. The 4*f* orbitals of rare-earth elements are shielded by outer 5*s* and 5*p* orbitals, leading to sharp and stable emission lines across the infrared, visible, and ultraviolet regions. Multiple rare-earth cations incorporated into a high-entropy lattice can enhance photoluminescence efficiency, increase defect tolerance, and broad the tunability of the emission spectra, which makes them suitable for applications in laser systems, phosphors, and next-generation display technologies [124]. Recent studies have demonstrated the application of rare-earth elements in optoelectronic applications through controlled synthesis and doping strategies [125]. Beyond traditional photoluminescence applications, RE-HEOs have also been explored for tunable bandgap engineering in optoelectronics. The incorporation of multivalent rare-earth cations such as Ce, Pr, and Tb into high-entropy fluorite-type oxides has enabled bandgap tuning through controlled synthesis atmospheres, broadening their applicability in optoelectronic devices. This tunability allows for the development of energy-efficient materials suitable for ultraviolet and visible-light photodetectors, as well as solar energy harvesting systems [122]. Studies on high-entropy zirconate-based oxides have shown remarkable phase stability and defect-tolerant photonic properties [126]. These materials maintain their structural integrity under high-radiation exposure but also exhibit a stable luminescent performance, which makes them suitable for deep-space exploration and high-energy optical sensors [126]. Another promising application of RE-HEOs in optoelectronics is in thermally stable phosphors for solid-state lighting. Conventional phosphor materials often suffer from thermal quenching, which degrades their luminescence efficiency at elevated temperatures. However, incorporating elements such as Eu, Gd, Dy, and Tb demonstrated reduced thermal quenching effects, maintaining a strong emission intensity even at temperatures exceeding 800 °C [127]. This characteristic is important for next-generation LED- and laser-based illumination systems [127]. Photoluminescence, the emission of light from a material upon absorption of photons, is fundamental in a variety of advanced applications, including solid-state lighting, displays, optical sensing, and quantum information technologies. Materials exhibiting strong, tunable photoluminescence have gained significant attention due to their potential to enhance efficiency, stability, and functionality in optoelectronic devices. Rare-earth-based materials, particularly oxides, are interesting materials for photoluminescent applications due to their unique 4f electronic configurations, which result in sharp emission lines and long-lived excited states [128]. The development of high-entropy materials with rare-earth elements ensures that the materials maintain their optical integrity under various operating conditions, making them candidates for long-term applications in optoelectronics and phosphors. The disorder induced by the entropy effect contributes to broadening the emission spectra while maintaining a high quantum efficiency [129]. The approach to modifying the photoluminescent behavior of rare-earth elements involves the doping of multiple rare-earth ions, such as Eu^3+^, Tb^3+^, and Dy^3+^, within a stable host matrix. Eu^3+^ ions, for example, are well known for their sharp red emissions due to electric dipole transitions, which makes them suitable for display technologies and lighting applications [130,131]. Tb^3+^ and Dy^3+^ ions, on the other hand, provide green and yellow emissions, respectively, facilitating multi-color luminescence within a single phase [132]. This compositional flexibility allows for tuning the emission spectra by varying the relative concentrations of different rare-earth ions, a significant advantage over traditional phosphor materials. Recent research showed that RE-HEOs based on zirconate and hafnate compositions exhibit remarkable photoluminescent properties due to their high thermal and chemical stability. In particular, pyrochlore-type RE_2_Zr_2_O_7_ compositions containing multiple rare-earth elements (La, Nd, Sm, Eu, Gd) displayed enhanced photoluminescence due to the cooperative effects of different luminescent centers and their interactions with the host lattice [133]. Thermal barrier coatings are an important factor in reducing thermal stresses and enhancing the lifespan of structural components in high-temperature applications, including jet engines, gas turbines, and hypersonic flight systems. Traditional TBC materials, such as yttria-stabilized zirconia, face significant limitations, including thermal conductivity degradation and phase instability beyond 1200 °C, which can be solved by the development of alternative compositions capable of maintaining structural integrity and thermal insulation at ultra-high temperatures [134,135]. Recent studies demonstrated that multicomponent zirconates with high configurational entropy can maintain their structural phase over prolonged thermal cycling [135]. Multicomponent pyrochlores, synthesized through entropy stabilization, exhibit enhanced thermal shock resistance and oxidation durability. Experimental results indicate that compositions such as (La_0.2_Ce_0.2_Pr_0.2_Sm_0.2_Eu_0.2_)_2_Hf_2_O_7_ can retain phase stability up to 1600 °C, outperforming traditional hafnates in high-temperature environments [136].

## 5. Future Perspectives

One of the most important challenges is the development of advanced synthesis techniques that are scalable and energy-efficient [137] while maintaining the structural and compositional integrity of RE-HEOs. The current methods are effective at the laboratory scale but require optimization to achieve industrial scalability. For example, Kumbhakar et al. [122] explored high-throughput synthesis and characterization approaches to accelerate the discovery and synthesis of novel RE-HEOs. Innovations in methods like flame spray pyrolysis and hydrothermal processing could allow for the production of RE-HEOs with high surface area, tailored defect concentration, and controlled particle sizes [138]. These scalable techniques are essential for expanding the use of RE-HEOs in commercial catalytic processes and energy storage systems. Recent advances in molten salt synthesis demonstrated that ultrafine RE-HEO powders with high crystallinity can be obtained at lower temperatures, reducing the energy costs and enabling their large-scale production [138]. However, challenges such as phase segregation, non-uniform elemental distribution, and stability under extreme conditions remain unsolved and require further investigation [126]. Most studies have been focused on a limited subset of rare-earth elements, often combined with transition metals [139]. Future research could systematically investigate the effects of incorporating less commonly studied rare-earth elements, such as terbium, dysprosium, and holmium, alongside unconventional dopants. Additionally, terbium and dysprosium have shown promise in magnetic and electronic applications, further proving the potential of RE-HEOs [140]. The strategic inclusion of these elements could further enhance the redox properties, thermal stability, and catalytic activity of RE-HEOs, enabling their application in more demanding environments, such as high-temperature fuel cells or CO_2_ hydrogenation under industrial conditions, and towards more precious products. Combining RE-HEOs with other functional materials could be also advantageous in the field of material science. Hybrid systems, integrating RE-HEOs with carbon-based materials such as graphene, carbon nanotubes, or porous carbons, could address challenges related to mass transport and active site dispersion [141]. Similarly, incorporating RE-HEOs within metal–organic frameworks or anchoring them to zeolite supports could enhance their selectivity and efficiency in catalytic reactions. These hybrid systems may also provide unique opportunities for the improvement of the local chemical environment around active sites, and their performance could be further improved in applications such as water splitting [98], thermochemical energy storage [142], and greenhouse gas reduction [22]. The defect chemistry of RE-HEOs, particularly the formation and dynamics of oxygen vacancies, significantly influences their catalytic [22] and electronic properties [143]. However, a deeper mechanistic understanding of how these vacancies interact with reactants during catalytic processes remains necessary. Advanced characterization techniques, such as operando X-ray absorption spectroscopy, neutron scattering, and in situ electron microscopy, should be employed to study vacancy formation, migration, and annihilation in real time for each composition [139]. Coupling these experimental approaches with computational modeling and machine learning-based simulations could provide new insights into the structure–function relationships characterizing RE-HEOs for industrial processes. Scalability and cost reduction will be two of the most important factors when determining the industrial relevance of RE-HEOs [144]. The relatively high cost of rare-earth elements and the energy-intensive nature of some synthesis methods are potential barriers to their commercialization. Amon et al. [144] reported potential barrier breakers through their research into energy-efficient and cost-efficient production techniques, such as aluminothermic reduction. The environmental implications of RE-HEOs also are worth consideration. While their catalytic properties make them promising for CO_2_ reduction, CO oxidation, hydrogen production, and pollution removal, a comprehensive life cycle determination is necessary to evaluate their overall environmental impact. Such investigations would provide valuable data on the carbon footprint, resource consumption, and end-of-life recyclability of RE-HEOs. In energy systems, for example, RE-HEOs could serve as next-generation electrodes or electrolytes in solid oxide fuel cells, batteries, and supercapacitors, benefiting from their high ionic conductivity and thermal stability [145]. Their high-entropy structure enables enhanced stability and ionic transport, making them suitable for fuel cells [81,146]. In catalysis, their unique defect structures and multi-component nature make them ideal for developing selective, robust catalysts for industrial chemical processes, such as ammonia synthesis, methane reforming, and hydrocarbon oxidation. For example, high-entropy perovskite oxides showed significant improvements in their oxygen redox reactivity, which directly support their role in electrocatalysis [46]. Finally, interdisciplinary collaboration will be essential to advancing the field of RE-HEOs. Chemists, material scientists, physicists, and engineers will need to work together to address the challenges associated with designing, synthesizing, and the applicability of these materials. The integration of computational modeling, machine learning, and high-throughput screening techniques could significantly accelerate the discovery of novel RE-HEO compositions with enhanced properties.
